# AIDS-defining events among people living with HIV who have been under continuous antiretroviral therapy for more than one year, a German cohort study 1999–2018

**DOI:** 10.1007/s15010-024-02188-y

**Published:** 2024-02-21

**Authors:** Annemarie Pantke, Christian Kollan, Barbara Gunsenheimer-Bartmeyer, Björn-Erik Ole Jensen, Christoph Stephan, Olaf Degen, Dirk Schürmann, Tobias Kurth, Viviane Bremer, Uwe Koppe, the HIV-1 Seroconverter Cohort and the ClinSurv HIV Cohort, Heribert Knechten, Petra Panstruga, Keikawus Arasteh, Michael Rittweger, Hans Wesselmann, Nikolai Menner, Ulrich Bohr, Heiko Jessen, Arne B. Jessen, Hubert Schulbin, Sascha Brand, Jan Gumprecht, Beate Weninger, Heribert Hillenbrand, Heiko Karcher, Klaus Fischer, Dietmar Schranz, Mathias Vallée, Jukka Hartikainen, Stephan Grunwald, Jörg A. Claus, Claudia Thomas, Roland Grimm, Sarah Schoor, Christiane Cordes, Reinhold Schröder, Tobias Glaunsinger, Michael Rausch, Thomas Reineke, Gordon Weinberg, Manuel Bruhy, Siegfried Köppe, Peter Kreckel, Andreas Berger, Sinah Lindemann, Norbert H. Brockmeyer, Anja Potthoff, Kathrin van Bremen, Jürgen Rockstroh, Martin Hower, Claudia Bachmann, Petra Spornraft-Ragaller, Dieter Teichmann, Björn-Erik Ole Jensen, Falk Hüttig, Stefan Esser, Pia Schenk-Westkamp, Annette Haberl, Christoph Stephan, Susanne Usadel, Matthias Müller, Janina Trauth, Alan Chavez-Valladares, Gerd Deutschinoff, Burkhard Kreft, Danica Lange, Olaf Degen, Guido Schäfer, Andreas Plettenberg, Frieder Kuhlendahl, Dorothea Wiemer, Lavinia Biemann, Knud Schewe, Christian Hoffmann, Georg Behrens, Matthias Stoll, Benjamin T. Schleenvoigt, Mathias W. Pletz, Ansgar Rieke, Stephan Schneeweiß, Stefan Scholten, Mark Oette, Peter A. Arbter, Thomas Grünewald, Jeannine Weidemann, Ines Ruck, Bernd Claus, Martin Sprinzl, Peter R. Galle, Matthias P. Ebert, Roger Vogelmann, Johannes Bogner, Ulrike Hellerer, Antoniya Todorova, Claudia Traidl-Hoffmann, Birgit Mück, Ramona Pauli, Christoph D. Spinner, Jochen Schneider, Birgit Mück, Robert Baumann, Niels Schübel, Christiane Berning, Franz Audebert, A. Trein, E. Schnaitmann, Clemens Roll, Simone Marquardt, Georg Härter, Beate Grüner, Cengiz Güler, Steve Rößler, Dirk Schürmann, Marianne Warncke, Jürgen Rockstroh, Jan-Christian Wasmuth, Svetlana Hass, Björn-Erik Ole Jensen, Cecilie Feind, Stefan Esser, Pia Schenk-Westkamp, Christoph Stephan, Annette Haberl, Peter Schott, Andreas Plettenberg, Thore Lorenzen, Frieder Kuhlendahl, Axel Adam, Thomas Buhk, Stephan Fenske, Stefan Hansen, Christian Hoffmann, Michael Sabranski, Knud Schewe, Hans-Jürgen Stellbrink, Dennis Radzuweit, Alexander Mainka, Constantin Rickassel, Olaf Degen, Guido Schäfer, Robin Scheiter, Matthias Stoll, Steve Gerschmann, Renate Beider, Heinz-August Horst, Silke Trautmann, Gerd Fätkenheuer, Jörg Janne Vehreschild, Laura Hamacher, Lennart Nicksch, Johannes Bogner, Barbara Sonntag, Oliver Pullen, Carlos Fritzsche

**Affiliations:** 1 Department of Infectious Disease Epidemiology, Robert Koch Institute, Seestraße 10, 13353 Berlin, Germany; 2 Institute of Public Health, Charité—Universitätsmedizin Berlin, Berlin, Germany; 3 Department of Gastroenterology, Hepatology and Infectious Diseases, University Hospital Düsseldorf, Medical Faculty, Heinrich-Heine-University Düsseldorf, Düsseldorf, Germany; 4 Medical Department 2, Infectious Diseases Unit, University Hospital of Frankfurt, Frankfurt, Germany; 5 Clinic for Infectious Diseases, University Medical Center Hamburg-Eppendorf, Hamburg, Germany; 6 Department of Infectious Diseases, Respiratory Medicine and Critical Care, Charité—Universitätsmedizin Berlin, Berlin, Germany

**Keywords:** AIDS, HIV-related opportunistic infections, Antiretroviral therapy, Long-term care, Clinical surveillance, Cohort analysis

## Abstract

**Purpose:**

This study examined the characteristics, incidence and prognostic factors of the first AIDS-defining condition developed after more than one year of continuous antiretroviral therapy (ART) among people living with HIV (PLHIV).

**Methods:**

We used data from two multicentre observational cohorts of PLHIV in Germany between 1999 and 2018. Our outcome was the first AIDS-defining event that occurred during follow-up after more than one year of continuous ART. Descriptive analyses at ART initiation, at the time of the AIDS event and of the most frequently observed types of AIDS-defining illnesses were performed. We calculated the incidence rate (IR) per 1000 person-years (PY) and used a bootstrap stepwise selection procedure to identify predictors of the outcome.

**Results:**

A total of 12,466 PLHIV were included in the analyses. 378 developed the outcome, constituting an overall IR of 5.6 (95% CI 5.1–6.2) AIDS events per 1000 PY. The majority of PLHIV was virally suppressed at the time of the event. Oesophageal candidiasis and wasting syndrome were the most frequently diagnosed AIDS-defining illnesses. We found a low CD4 count at ART initiation, a previous AIDS-defining condition and transmission through intravenous drug use to be meaningful prognostic factors of the outcome.

**Conclusion:**

The overall rate of AIDS-defining events among PLHIV under long-term ART was low, highlighting the importance of continuous treatment. PLHIV who started ART with indicators of impaired immune functioning were more susceptible to disease progression, suggesting that the public health response should continue to focus on early and sustained treatment for all PLHIV.

**Supplementary Information:**

The online version contains supplementary material available at 10.1007/s15010-024-02188-y.

## Background

The introduction of combination antiretroviral therapy (ART) in 1996 has been a milestone in the treatment of HIV. Continuous improvements in ART efficacy and safety as well as adapted prescription guidelines have led to a substantial decline in HIV-related morbidity and mortality among people living with HIV (PLHIV) worldwide [1, 2]. In Germany as of 2021, 96% of all diagnosed PLHIV received HIV treatment of which another 96% had achieved durable viral suppression [3]. With regard to the Joint UN Program on HIV/AIDS (UNAIDS)’s postulated “95-95-95” targets that aim to have diagnosed 95% of all HIV-positive people, provided ART for 95% of those diagnosed and achieved viral suppression for 95% of those treated by 2030 [4], Germany has reached the two ART-related endpoints well ahead of the target date. On the basis of these goals, the overarching aim put forward by UNAIDS was to “end the AIDS epidemic by 2030” [4].

These encouraging developments are reflected in the incidence rates (IRs) of AIDS in Germany. In a previous study, we have analysed changes in the AIDS rates among PLHIV under clinical care by years of follow-up as well as calendar periods between 1999 and 2018 [5]. In line with other prior research conducted in the Western European and North American context, we found that the IR of a first AIDS-defining condition has continuously declined by both years of follow-up and calendar periods since the introduction of effective ART [5–8]. The majority of AIDS events were observed at baseline and within the first year of follow-up, suggesting that the progression to AIDS can largely be attributed to late HIV diagnoses [5]. This was further corroborated by our finding that indicators of advanced HIV infections, i.e., a low CD4 cell count, high viral load, and older age at baseline, were predictive of disease progression [5].

Despite these developments, there still remains a number of PLHIV in Germany who progress to AIDS even after long-term ART exposure [5]. Even though these cases can be considered rare since the advent of effective ART [9], it is important to examine them since their occurrence can neither be attributed to a still recovering immune response as AIDS events in the early phase after starting ART nor initial complications like the development of the immune reconstitution inflammatory syndrome (IRIS), a hyperinflammatory reaction that usually manifests within the first months after starting treatment [10, 11]. The incidence and specific characteristics of AIDS events developed after continuous long-term therapy in the era of effective ART have not been sufficiently examined yet.

The objective of the current study was, therefore, to analyse the occurrence of AIDS-defining illnesses among PLHIV with long-term regular ART intake from two German observational cohorts. More specifically, we were interested in examining the incidence of the first AIDS-defining condition developed after more than one year of continuous ART. Here, we aimed to analyse the characteristics of these PLHIV at the time of ART initiation, at the time of the AIDS event as well as the types of AIDS-defining illnesses that were diagnosed. In addition to that, we sought to identify potential predictors of the first AIDS-defining condition that occurred under regular ART. A better understanding of those AIDS cases is needed to assess the feasibility of UNAIDS’s goal of “ending the AIDS epidemic by 2030” [4] in the context of Germany and can help to direct the public health response on the route to achieving this goal.

## Methods

### Study design and population

We used data from the HIV-1 Seroconverter Study and ClinSurv HIV Study, which are two German multicentre, open, prospective long-term observational cohorts initiated in 1997 and 1999, respectively [12, 13]. Both cohorts are hosted and coordinated at the Robert Koch Institute (RKI) where the data are used for the clinical surveillance of PLHIV in Germany. Participating HIV practitioners regularly report pseudonymised information on demographic properties, laboratory values, and other clinical events from routine visits. In the HIV-1 Seroconverter Study, eligibility criteria include the knowledge of a person’s approximate time of infection based on a negative HIV test within three years before the diagnosis or a diagnosis during acute seroconversion. In the ClinSurv HIV Study, all PLHIV who receive treatment in one of the participating HIV clinics are eligible for inclusion. The study designs have been described in detail in previous publications [5, 12, 13]. In the current study, we combined the data of both cohorts and included PLHIV who were enrolled between 1999 and 2018. We excluded PLHIV under the age of 18 years and PLHIV whose ART initiation date was missing or who had initiated ART before the enrolment in one of the cohorts. In the case of ART interruptions, we censored all patient observations that were recorded after the first interruption to only include person-time of PLHIV while being prescribed ART. We furthermore excluded all PLHIV from the analyses who did not complete a year of ART, which includes those who developed an AIDS event within the first year after ART initiation.

### Outcome

Our outcome was the first AIDS-defining event that occurred during follow-up after more than one year of continuous ART since therapy initiation. An AIDS-defining event was comprised of a diagnosis of one or potentially more AIDS-defining illnesses in the same month as the cohort data are recorded monthly. We included all clinical conditions associated with AIDS as published by the Centers for Disease Control and Prevention (CDC) [14]. As opposed to the CDC’s expanded AIDS surveillance case definition, we did not characterise a CD4 cell count drop below 200 cells/µL as an AIDS-defining event [14].

### Predictor selection

We selected potential predictors of the first AIDS-defining event developed after more than one year of continuous ART on the basis of prior research and availability in our dataset [15–18]. These included age at ART initiation, gender, transmission mode, country of origin, CD4 count at ART initiation, plasma HIV-1 RNA viral load (VL) at ART initiation, and a diagnosis of a previous AIDS event before or at ART initiation. Age, CD4 count and VL were treated as continuous variables. Gender was categorised into male vs. female, transmission mode into men who have sex with men (MSM), persons with heterosexual contact (HET), persons who inject drugs (PWID), persons from high-prevalence countries (PHPC) and “other”, country of origin into Germany vs. abroad, and a previous AIDS event into yes vs. no. The transmission group PHPC comprised PLHIV who migrated from countries with an HIV prevalence of > 1% among the population between 15 and 49 years.

### Statistical analysis

We performed descriptive analyses at baseline, i.e., the month of ART initiation. The ART initiation date could fall on the same date as a person’s enrolment date in one of the two cohorts or later if ART was prescribed over the course of follow-up. We present the mean and standard deviation (SD) for the variable age and the median and interquartile range (IQR) for CD4 count and VL. In the case of missing CD4 or VL values at baseline, we used, if available, records up to three months after ART initiation. For the categorical variables, percentages are presented and corresponding 95% confidence intervals (95% CI) when proportions are described and compared. To examine whether the baseline data differed among PLHIV who experienced the outcome at different points in time throughout follow-up, we additionally stratified the baseline characteristics by years under continuous ART at the time of the AIDS event. The strata selected were AIDS after > 1–3 years, > 3–6 years, and > 6 years of continuous ART, with 3 years corresponding to the approximate median time and 6 years to the third quartile until the outcome.

We performed further descriptive analyses of the characteristics at the time of the first AIDS event under continuous ART. Here, we present the time-varying variables age, CD4 count and VL. In addition to that, we created the variable “Experienced viral load increase (> 200 copies/mL) before the AIDS event”, which indicated the occurrence of at least one episode of viraemia before the AIDS event after already having achieved viral suppression. Since a detectable VL at the time of the AIDS event can be an indicator of suboptimal ART adherence [19], we additionally stratified VL by transmission mode to examine potential differences between the groups. As in our baseline analyses, we also performed stratified analyses by the time until the AIDS event occurred.

Further descriptive analyses were performed with regard to the types of AIDS-defining illnesses. We present the five most frequently observed AIDS-defining illnesses among PLHIV who developed the AIDS event after more than one year of continuous ART intake. To further analyse whether certain illnesses were diagnosed more often at particular CD4 or VL categories, differed across the transmission groups or with respect to the time under continuous ART until the AIDS event, we also stratified the analyses of the AIDS-defining illnesses on these variables. We do not present the transmission groups “other” and “unknown” due to sparse data and limited interpretability.

In our time-to-event analyses, we first calculated the incidence rate (IR) and corresponding 95% CI of the first AIDS-defining event per 1000 person-years (PY). Follow-up started one year after ART initiation. PLHIV were censored at the first AIDS-defining event, the first ART interruption, loss-to-follow-up, death or administrative censorship (December 31, 2018). Seeing that with the continuous improvements in ART efficacy and safety treatment regimens and guidelines were amended over time, we split our study period and additionally calculated the IR in each period for comparison. We selected the time periods of 1999 to 2009 and 2010 to 2018, including only person-time of PLHIV within the respective period. 2010 was chosen as the dividing year as the US Department of Health and Human Services (DHHS) and the German and Austrian AIDS societies (DAIG, ÖAG) amended their guidelines at that time, recommending ART generally for all patients with CD4 counts below 500 cells/µL [20, 21].

To identify prognostically meaningful variables for the development of an AIDS event, we utilised the bootstrap stepwise selection procedure by Sauerbrei and Schumacher [22]. Here, we carried out 500 bootstrap replications of the original data set. In each replication, potential predictors were selected by a stepwise algorithm which only kept variables in the Cox proportional hazards regression model that did not exceed our a priori selected cut-off *p*-value of 0.1. The absolute and relative inclusion frequencies then served as a criterion for the predictive importance of the respective variables. We considered variables as predictive that were selected in the model in a high majority (> 80%) of the bootstrapped samples. As our potential predictors were all continuous or binary except for transmission mode, we dichotomised transmission mode and conducted sensitivity analyses repeating the selection procedure with separate binary variables for each transmission group. MSM served as the reference category for transmission mode and was not included as a variable for collinearity reasons. The transmission groups “other” and “unknown” were excluded due to sparse data and limited interpretability. This sensitivity check was conducted to examine whether one or more of the transmission groups stood out as a meaningful predictor of the outcome. To detect potential time trends, we also carried out the predictor analyses in each of our selected time periods (1999–2009 and 2010–2018).

All analyses were performed using Stata 17.0 (Stata Statistical Software: Release 17, United States).

### Ethical approval

The HIV-1 Seroconverter Study was approved by the ethics commission at the Charité—Universitätsmedizin Berlin (EA2/105/05). The ClinSurv HIV Study data between 1999 and 2018 were collected anonymously in compliance with the German Infection Protection Act (IfSG) as of 2001. As the data collection adhered to the legal requirements of the IfSG, no written informed consent was required. Approval was granted by the RKI data protection officer and the Federal Commissioner for Data Protection and Freedom of Information.

## Results

### Baseline characteristics

**Fig. 1:**
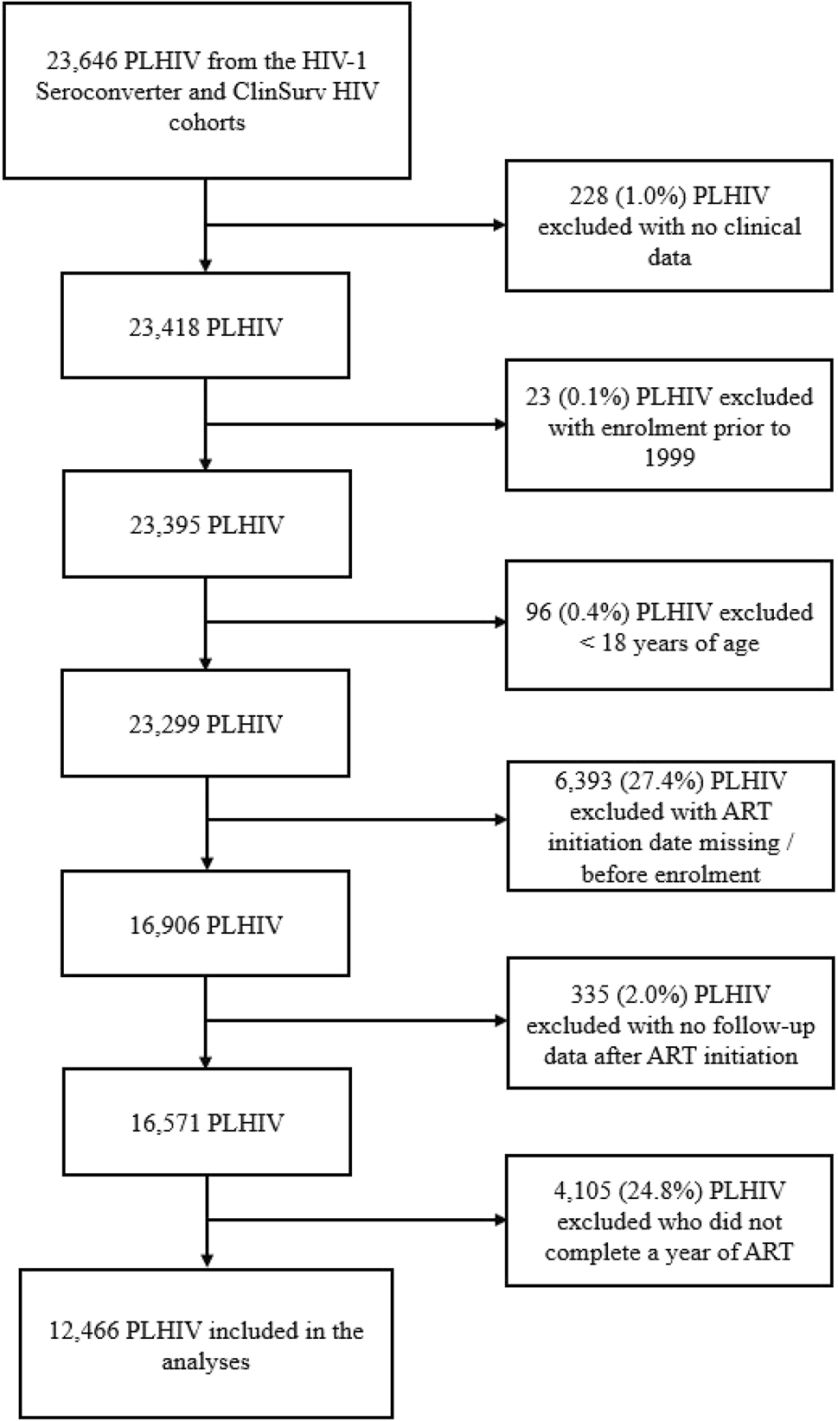
Flowchart of people living with HIV (PLHIV) included in the analyses, selected from the German HIV-1 Seroconverter and ClinSurv HIV cohorts

A total of 12,466 PLHIV were included in the analyses (Fig. 1), of which 378 experienced an AIDS-defining event after more than one year of continuous ART and 12,088 did not. Table 1 presents the baseline characteristics of both groups, showing that they were comparable with regard to age, gender, country of origin, and VL. Mean age at ART initiation was 40.3 years (SD 10.6) among PLHIV with the outcome and 39.9 years (SD 10.8) among PLHIV without the outcome. The majority of PLHIV were male (80.4%, 95% CI 76.1–84.3 vs. 80.8%, 95% CI 80.1–81.5) and born in Germany (68.8%, 95% CI 63.8–73.4 vs. 68.9%, 95% CI 68.0–69.7). While the transmission groups HET (18.0%, 95% CI 14.2–22.2 vs. 15.3%, 95% CI 14.7–16.0) and PHPC (15.3%, 95% CI 11.9–19.4 vs. 13.3%, 95% CI 12.7–13.9) were comparable in size, MSM were less frequently represented among PLHIV who developed an AIDS-defining condition (45.8%, 95% CI 40.7–50.9 vs. 52.1%, 95% CI 51.2–53.0) and PWID more frequently (10.6%, 95% CI 7.7–14.1 vs. 5.3%, 95% CI 4.9–5.7). PLHIV with an AIDS event had lower CD4 counts at ART initiation compared to those without an AIDS event (median 178 cells/µL, IQR 73–313 vs. median 280 cells/µL, IQR 144–433), while the VLs were comparable (median 24,930 copies/mL, IQR 350–164,313 vs. median 10,000 copies/mL, IQR 219–110,000). Moreover, PLHIV with the outcome more often had already experienced a previous AIDS-defining condition at baseline compared to those without the outcome (32.3%, 95% CI 27.6–37.2 vs. 14.6%, 95% CI 14.0–15.3).

**Table 1:** Baseline characteristics of PLHIV who have been under continuous ART for > 1 year

	PLHIV who experienced an AIDS event *n* (%)	PLHIV without an AIDS event *n* (%)
Total	378 (100.0%)	12,088 (100.0%)
Age (years)		
Mean (SD)	40.3 (10.6)	39.9 (10.8)
18–29	63 (16.7%)	2168 (17.9%)
30–39	129 (34.1%)	4149 (34.3%)
40–49	122 (32.3%)	3552 (29.4%)
50–59	42 (11.1%)	1578 (13.1%)
60–69	18 (4.8%)	531 (4.4%)
> 69	4 (1.1%)	110 (0.9%)
Gender		
Male	304 (80.4%)	9764 (80.8%)
Female	74 (19.6%)	2324 (19.2%)
Transmission mode		
Men who have sex with men	173 (45.8%)	6300 (52.1%)
Persons with heterosexual contact	68 (18.0%)	1854 (15.3%)
Persons who inject drugs	40 (10.6%)	636 (5.3%)
Persons from high-prevalence countries	58 (15.3%)	1610 (13.3%)
Other	0 (0.0%)	78 (0.7%)
Unknown	39 (10.3%)	1610 (13.3%)
Country of origin		
Germany	260 (68.8%)	8322 (68.9%)
Abroad	115 (30.4%)	3496 (28.9%)
Unknown	3 (0.8%)	270 (2.2%)
CD4 count (cells/µL)		
Median (IQR)	178 (73–313)	280 (144–433)
< 50	59 (15.6%)	1107 (9.2%)
50–199	114 (30.2%)	2559 (21.2%)
200–499	107 (28.3%)	4993 (41.3%)
> 500	35 (9.3%)	1928 (16.0%)
Missing	63 (16.7%)	1501 (12.4%)
Viral load (copies/mL)		
Median (IQR)	24,930 (350–164,313)	10,000 (219–110,000)
< 50	35 (9.3%)	1624 (13.4%)
50–999	62 (16.4%)	2162 (17.9%)
1000–9999	39 (10.3%)	1398 (11.6%)
10,000–99,999	58 (15.3%)	2414 (20.0%)
> 100,000	105 (27.8%)	2787 (23.1%)
Missing	79 (20.9%)	1703 (14.1%)
Previous AIDS event^a^		
Yes	122 (32.3%)	1770 (14.6%)
No	256 (67.7%)	10,318 (85.4%)

Numbers may not add up to 100% because of rounding

*PLHIV* People living with HIV *ART* Antiretroviral therapy *SD* Standard deviation *IQR* Interquartile range

^a^A previous AIDS event includes AIDS-defining illnesses that were diagnosed after enrolment and before or at the time of ART initiation

Our stratified analyses of PLHIV with an AIDS event showed that in the majority the event occurred after > 1–3 years since ART initiation (Table S1, Additional file 1). PLHIV who experienced the outcome after > 6 years were predominantly male (91.1%, 95% CI 83.2–96.1). CD4 counts at baseline were higher among PLHIV who developed an AIDS event later during follow-up (> 1–3 years: median 153 cells/µL, IQR 40–296, > 3–6 years: median 181 cells/µL, IQR 85–298, > 6 years: median 214 cells/µL, IQR 119–362).

### Characteristics at the time of the AIDS-defining event

In Table 2, we present the characteristics at the time of the AIDS event among the 378 PLHIV who experienced the outcome. We observed a mean age of 44.6 years (SD 11.1) and a median CD4 count of 324 cells/µL (IQR 127–510). With regard to the CD4 counts and VLs, missing values were obtained in more than half of the cases. 66.3% of the PLHIV with available VL records were virally suppressed (< 50 copies/mL) when the AIDS-defining condition was diagnosed. Over 90% had achieved viral suppression over the course of follow-up before the AIDS event. Here, 58.5% had sustained durable viral suppression or experienced only minor VL increases at some point before the AIDS event, and 32.0% had experienced at least one episode of viraemia with more than 200 copies/mL. 9.5% had never achieved viral suppression before the event occurred (Table 2). In the additional stratified analyses of VL, we observed the lowest proportion of viral suppression at the time of the AIDS event among PWID (42.9%, 95% CI 17.7–71.1) (Table S2, Additional file 2).

**Table 2:** Characteristics at the time of the AIDS event among PLHIV who experienced an AIDS-defining event after > 1 year of continuous ART

Total	378 (100.0%)
Age (years) at the time of the AIDS event	
Mean (SD)	44.6 (11.1)
18–29	26 (6.9%)
30–39	108 (28.6%)
40–49	132 (34.9%)
50–59	73 (19.3%)
60–69	29 (7.7%)
> 69	10 (2.7%)
CD4 count (cells/µL) at the time of the AIDS event	
Median (IQR)	324 (127–510)
< 50	21 (12.1%)
50–199	42 (24.3%)
200–499	65 (37.6%)
> 500	45 (26.0%)
Missing^a^	205
Viral load (copies/mL) at the time of the AIDS event^b^	
< 50	112 (66.3%)
50–999	18 (10.7%)
1000–9999	11 (6.5%)
10,000–99,999	11 (6.5%)
> 100,000	17 (10.1%)
Missing^a^	209
Experienced viral load increase (> 200 copies/mL) before the AIDS event	
Yes	121 (32.0%)
No	221 (58.5%)
Never achieved viral suppression	36 (9.5%)

Numbers may not add up to 100% because of rounding

*PLHIV* People living with HIV *ART* Antiretroviral therapy *SD* Standard deviation *IQR* Interquartile range

^a^Due to the high number of missing values in CD4 count and viral load, percentages were calculated excluding missing values

^b^As the majority of PLHIV with available viral load values was virally suppressed at the time of the AIDS event, the presentation of a median viral load is not informative

Our stratified analyses by follow-up time showed that the later throughout follow-up the AIDS-defining condition developed, the higher the age (> 1–3 years: mean 41.1 years, SD 10.2, > 3–6 years: mean 46.3 years, SD 12.2, > 6 years: mean 49.3 years, SD 9.3). CD4 counts at the time of the AIDS event were highest among PLHIV who experienced the outcome after > 6 years of continuous ART (median 397 cells/µL, IQR 139–738). We observed no discernible pattern with regard to the proportion of PLHIV with undetectable VLs (> 1–3 years: 66.3%, 95% CI 55.5–76.0, > 3–6 years: 65.9%, 95% CI 49.4–79.9, > 6 years: 66.7%, 95% CI 49.8–80.9) (Table S3, Additional file 2). While 16.7% (95% CI 11.5–22.9) of the PLHIV who developed an AIDS condition after > 1–3 years had experienced at least one episode of viraemia above 200 copies/mL at some point before the outcome, this proportion increased to 64.4% (95% CI 53.7–74.3) among PLHIV who experienced the outcome after > 6 years (Table S3, Additional file 2).

### Types of AIDS-defining illnesses

The five most frequently observed AIDS-defining illnesses were oesophageal candidiasis (14.7%), wasting syndrome (11.3%), pneumocystis-jirovecii-pneumonia (10.3%), HIV encephalitis (10.1%), and HSV ulcers (7.5%) (Table 3).

**Table 3:** Five most frequently observed AIDS-defining illnesses among PLHIV who experienced an AIDS-defining event after > 1 year of continuous ART

AIDS-defining illness	*n* (%)
Total^a^	416 (100.0%)
Oesophageal candidiasis	61 (14.7%)
Wasting syndrome	47 (11.3%)
Pneumocystis-jirovecii-pneumonia	43 (10.3%)
HIV encephalitis	42 (10.1%)
HSV ulcers	31 (7.5%)

*PLHIV* People living with HIV *ART* Antiretroviral therapy

^a^The number of recorded AIDS-defining illnesses exceeds the number of first AIDS-defining events since an AIDS event can comprise more than one AIDS-defining illness if these were recorded in the same month

Several differences were found in the stratified analyses: Among PLHIV who developed the AIDS event after > 1–3 years of continuous ART, we observed oesophageal candidiasis (13.8%), wasting syndrome (10.8%), pneumocystis-jirovecii-pneumonia (9.7%), as well as extrapulmonary tuberculosis (9.2%) and pulmonary tuberculosis (8.2%) (Table S3, Additional file 3). While oesophageal candidiasis, wasting syndrome, and pneumocystis-jirovecii-pneumonia were also among the five most common illnesses of PLHIV who experienced the outcome after > 6 years, the tuberculoses were replaced by HIV encephalitis (13.0%) and atypical mycobacteria (8.0%). With respect to the transmission groups, we observed extrapulmonary tuberculosis and pulmonary tuberculosis only among the top five illnesses of PHPC with 25.0% and 11.7%, respectively, while the diagnosed illnesses among MSM, HET, and PWID were comparable and predominantly included wasting syndrome and oesophageal candidiasis. The types of illnesses also differed somewhat with regard to the CD4 counts at which they developed. Cerebral toxoplasmosis and cytomegalovirus manifested more frequently at lower CD4 counts (< 50 cells/µL: both 9.4%, 50–199 cells/µL: both 8.2%), while HSV ulcers and Kaposi’s sarcoma more frequently at higher CD4 counts of > 500 cells/µL (17.0% and 10.6%). No discernible patterns were found with regard to the VLs (Table S3, Additional file 3).

### Incidence and predictors

After completing a year of ART, a total of 12,466 PLHIV contributed person-time and were followed-up for a median of 4.3 years (range 1 month to 18.8 years). We recorded 378 first AIDS-defining events that constituted an IR of 5.6 (95% CI 5.1–6.2) AIDS events per 1000 PY among PLHIV under continuous ART between 1999 and 2018. Median follow-up time until the AIDS event was 2.2 years (range 1 month to 17.2 years), which translates into a median time of 3.2 years (range 13 months to 18.2 years) from ART initiation until the AIDS event. In our stratified time period analyses, we obtained an IR of 11.1 (95% CI 9.6–12.8) AIDS events per 1000 PY between 1999 and 2009 and an IR of 3.9 (95% CI 3.1–4.8) between 2010 and 2018.

In Table 4, we present the absolute and relative inclusion frequencies of the potential predictors after 500 bootstrap replications. The variables CD4 count at ART initiation and a previous AIDS event before ART initiation were included in the model in 499 (99.8%) and 498 (99.6%) of the bootstrapped samples, suggesting that these two variables were strong prognostic factors for the development of an AIDS-defining condition after more than one year of continuous ART. The direction of the predictors was derived from our descriptive analyses, which indicated that the lower the CD4 count at baseline and “yes” in regard to a previous AIDS event, the higher the risk of developing an AIDS-defining condition (Table 1). The variables country of origin, transmission mode, gender, VL at ART initiation, and age at ART initiation followed with inclusion frequencies of 176 (35.2%), 170 (34.0%), 79 (15.8%), 55 (11.0%), and 43 (8.6%) times, respectively, suggesting that neither of those were of meaningful predictive importance for the outcome. While we observed a similar distribution of the variables in the time period between 1999 and 2009 (Table S5, Additional file 4), none of the variables were selected in > 80% of the bootstrapped samples in the later period between 2010 and 2018 (Table S6, Additional file 4).

**Table 4:** Bootstrap replication inclusion frequencies of potential predictors for an AIDS-defining event among PLHIV after > 1 year of continuous ART

Covariate	Absolute inclusion frequency^a^	Relative inclusion frequency	
CD4 count (cells/µL) at ART initiation	499		99.8%
Previous AIDS event before ART initiation	498		99.6%
Country of origin	176		35.2%
Transmission mode^b^	170		34.0%
Gender	79		15.8%
Viral load (copies/mL) at ART initiation	55		11.0%
Age at ART initiation	43		8.6%

*PLHIV* People living with HIV *ART* Antiretroviral therapy

^a^Absolute inclusion frequencies after 500 bootstrap replications of the original data set

^b^Transmission groups “other” and “unknown” were excluded from the analyses due to sparse data and limited interpretability

Our sensitivity analyses in which we repeated the bootstrap stepwise selection procedure with separate binary variables for each transmission mode category showed that again, CD4 count at ART initiation and a previous AIDS event before ART initiation were selected in nearly all of the bootstrapped samples (Table S7, Additional file 4). In addition to that, the transmission group PWID was included in the model 407 (81.4%) times, suggesting that intravenous drug use can be considered another relevant predictor of the development of an AIDS-defining condition while receiving ART. HET and PHPC were included in only 156 (31.2%) and 103 (20.6%) of the bootstrap replications and were therefore not considered predictive of the outcome. In our time period analyses, we again observed a similar distribution in the first period between 1999 and 2009, in which a previous AIDS event and CD4 count were selected in the majority of the bootstrapped samples, however not PWID (Table S8, Additional file 4). In the second period between 2010 and 2018, again no variable crossed the 80% mark, suggesting that none of these variables were of meaningful predictive utility of the outcome in the later time period (Table S9, Additional file 4).

## Discussion

This study investigated the characteristics, incidence and prognostic factors of a first AIDS-defining condition developed after more than one year of continuous ART among PLHIV in Germany between 1999 and 2018. Our main findings were that PLHIV with the outcome had lower CD4 counts at ART initiation, had more often already developed an AIDS event before ART was initiated and more often were intravenous drug users compared to those without the outcome. At the time of the AIDS event, the majority of PLHIV was virally suppressed and nearly 60% had sustained durable viral suppression or experienced only minor VL increases at some point before the event. In addition to that, we found that oesophageal candidiasis and wasting syndrome were the most frequently diagnosed AIDS-defining illnesses. While these were observed across all strata, other illnesses occurred more often among certain strata such as extrapulmonary and pulmonary tuberculosis, which were diagnosed more frequently among people from high-prevalence countries. We obtained an overall low IR of the outcome, which was lower in the later study period between the years 2010 and 2018 compared to the earlier period between 1999 and 2009. A low CD4 count, a previous AIDS-defining condition at ART initiation and transmission through intravenous drug use were identified as overall meaningful prognostic factors of the outcome.

What stood out in our descriptive analyses was the observation that the majority of PLHIV with available VL records was virally suppressed at the time of the AIDS event. This finding builds on existing evidence of prior studies, which have also reported diagnoses of AIDS-defining conditions in the presence of undetectable VLs [23–26]. While the proportion of virally suppressed PLHIV at the time of the AIDS event was the same across the time strata, the proportion of PLHIV who experienced at least one episode of viraemia at some point before the AIDS event increased substantially the later across follow-up the event occurred. Even though we only analysed person-time from patient records indicating continuous ART intake, occasional non-adherence which can lead to episodes of viraemia can happen due to various reasons [27, 28]. In addition to that, although rare, HIV drug resistance resulting in viral failure can emerge despite potent ART drugs and adherence [29]. We assume that both sporadic ART non-adherence and drug resistance could have played a role in our study population, seeing that we also observed a considerable number of PLHIV with high VLs at the time of the AIDS event. The fact that even short episodic viraemia can negatively affect the immune response [26] highlights the importance of supporting ART adherence and a close monitoring of the VLs to spot potential fluctuations.

Insights into further potential factors to be regarded over the course of treatment are provided by our findings of the analysis of the most frequently diagnosed AIDS-defining illnesses. The results were partially consistent with those of our previous study in which we had analysed the AIDS rates among PLHIV under clinical care irrespective of ART status or duration [5]. While oesophageal candidiasis, wasting syndrome, and pneumocystis-jirovecii-pneumonia were also found among the top five illnesses in our previous study, HIV encephalitis and HIV ulcers as in the present study were not. As our stratified analyses showed, both illnesses, particularly HIV encephalitis, developed more often later in time after several years of continuous ART. Previous research found that certain diseases such as cardiovascular diseases and diabetes can increase the risk of HIV encephalitis [30]. Seeing that such noncommunicable conditions which are widespread and age-related [31, 32] potentially increase the risk of AIDS-defining conditions emphasises that attention also needs to be given to the comorbidities of PLHIV in therapy.

This is further corroborated by our finding that tuberculosis was the most common illness only among migrants from high-prevalence countries. As pointed out by prior research, the prevalence of the different types of AIDS-defining illnesses differs by region with tuberculosis disproportionally affecting countries in Sub-Saharan Africa [33–35], which make up the majority of the high-prevalence countries [36]. Besides HIV being a prognostic factor of tuberculosis itself, other health-affecting factors that are more common in these regions such as lower socioeconomic status together with malnutrition, improper housing conditions, or the usage of contaminated water further increase the risk of tuberculosis [37–39]. This finding hence also underlines the significance of PLHIV’s overall health status in regard to disease progression, which should be regularly checked and strengthened.

The results of our prediction analysis provide new insights into certain prognostic characteristics at ART initiation. The most often selected predictors for the development of an AIDS event after more than one year of continuous treatment were a low CD4 count and a previous AIDS-defining condition, which are both indicators of advanced HIV infections and an already substantially impaired immune response [3, 40]. While our and other previous studies that considered all AIDS cases including those developed shortly after ART initiation reported additional predictors such as a high VL and older age [5, 15–18], the findings of the present study suggest that factors directly related to immune functioning are the most critical when it comes to AIDS events after continuous long-term ART exposure. Our stratified analyses by time period revealed that while these two factors were also the strongest predictors in the years between 1999 and 2009, none of our available potential predictors showed to be of meaningful predictive importance in the later time period between 2010 and 2018. This finding suggests that with improved therapy regimens and amended guidelines recommending earlier treatment, these factors at ART initiation have become less useful to predict the occurrence of AIDS events after continuous long-term therapy. Further analyses considering additional variables would be needed to identify relevant prognostic factors among PLHIV who have initiated treatment in the more recent years.

Even though the variable transmission mode was not predictive of the outcome and therefore has only a little importance in the clinical context when considering prognostic factors, our sensitivity analyses of the entire study period showed that one category stood out and was predictive, which was transmission through intravenous drug use. In line with other prior research, this supports the point that PWID represents a vulnerable population with a higher susceptibility to disease progression after long-term treatment, potentially due to additional challenges with respect to treatment adherence, overall health, and access to clinical care with a resulting higher risk of late diagnoses [41–44]. Our analyses have also shown that PWID were the group that most often had detectable VLs at the time of the AIDS event, possibly indicating suboptimal adherence. Adherence issues among PWID can be explained by social limitations such as homelessness which are observed at higher rates among this population [45]. In addition to that, PWID more often experience mental health disorders that can affect treatment adherence and are overall at an increased risk of developing various comorbidities [46, 47], making them more susceptible to HIV disease progression.

Overall, the results of our study are encouraging and confirm the substantial improvements with respect to HIV-related morbidity among PLHIV in therapy since the advent of effective ART. In our 20-year study period, we observed only 378 PLHIV with a first AIDS-defining event under continuous long-term ART that constituted an overall low rate which was even lower in the more recent study period, highlighting the importance of sustained clinical care and ART. The main objective therefore remains to ensure that all HIV positive persons are diagnosed early and receive continuous treatment, which is underlined by our finding that indicators of advanced HIV infections at the time of ART initiation were overall strong prognostic factors of the outcome. Even though the rate was low, our results however also demonstrated that AIDS-defining events among PLHIV under long-term ART still occur, and they do so across a broad time span, with a wide range of different types of illnesses as well as in the presence of viral suppression. This allows for the assumption that even if all UNAIDS “95-95-95” targets were met or exceeded, there would still remain a small number of AIDS cases. To advance towards UNAIDS’s goal of “ending the AIDS epidemic by 2030” [4], we therefore suggest that the public health response continues to focus on early HIV detection to minimise the number of late diagnoses and consequently the number of PLHIV starting treatment with impaired immune functioning. In addition to that, supporting ART adherence and closely monitoring HIV-related parameters remains of importance, in particular among PLHIV who are more susceptible to disease progression.

### Strengths and limitations

Strengths of the present study include the long observation time as well as the large number of PLHIV included in the analyses. As our study period comprised almost the entire time of treatment with effective ART since its introduction, our results can be considered informative for Germany and other countries with similar characteristics in terms of ART coverage, HIV prevalence and other population demographics.

Limitations of our study include the substantial proportion of missing CD4 and VL values at the time of the AIDS event. This might have led to biased results with a potential shift in the distribution of these variables, which would have particularly affected the conclusions in regard to PLHIV with undetectable VLs at the time of the AIDS event. Since our stratified analyses however showed similar proportions of missing CD4 and VL values across the time strata and we have no further knowledge of a certain pattern regarding PLHIV whose values at the time of the outcome were measured or not, we assume that the overall results were not considerably affected by the missing data. Another limitation to be considered is that the participating HIV clinics are mostly specialised centres in which a higher number of PLHIV with advanced HIV infections is treated [13]. We might have therefore obtained somewhat higher IRs compared to the ones that would have been observed in the entire population of PLHIV in Germany. Finally, the common limitations of cohort studies also apply to our study, such as loss to follow-up including drop-outs that potentially resulted in missed outcomes and attrition bias.

## Conclusion

Our study has shown that the overall rate of a first AIDS-defining event among PLHIV who have been receiving continuous ART for more than a year was low, highlighting the importance of sustained clinical care and ART. Nevertheless, AIDS events after long-term therapy still occur in the era of effective ART and can be expected across a broad time span as well as with different types of AIDS-defining illnesses. Indicators of impaired immune functioning at treatment initiation, i.e., a low CD4 count, a previous AIDS-defining condition and transmission through intravenous drug use, showed to be the most meaningful prognostic factors. Overall, our findings suggest that to further reduce the number of AIDS events among PLHIV receiving ART, the public health response should continue to focus on ensuring early and sustained treatment for all PLHIV.

### Supplementary Information

Below is the link to the electronic supplementary material.

Supplementary file1 (DOCX 18 KB)

Supplementary file2 (DOCX 16 KB)

Supplementary file3 (DOCX 16 KB)

Supplementary file4 (DOCX 15 KB)

## Data Availability

The data generated and/or analysed during the current study are not publicly available due to data protection and confidentiality agreements.
