# Supplementary material for: AIDS-defining events among people living with HIV who have been under continuous antiretroviral therapy for more than one year, a German cohort study 1999–2018
Source: Infection. 2024 Feb 21;52(2):637–48. doi: 10.1007/s15010-024-02188-y (PMC10954987; doi:10.1007/s15010-024-02188-y)
Supplement: Supplementary file 1 — Supplementary file1 (DOCX 18 KB) [file 15010_2024_2188_MOESM1_ESM.docx]

| **Additional file 1: Table S1**  **Table S1 Baseline characteristics of PLHIV who experienced an AIDS-defining event after > 1 year of continuous ART**  Stratified by years under continuous ART at the time of the AIDS event | | | |
| --- | --- | --- | --- |
| **Years under continuous ART**  **at the time of the AIDS event** | **> 1 to 3 *n* (%)** | **> 3 to 6 *n* (%)** | **> 6 *n* (%)** |
| Total | 180 (100.0%) | 108 (100.0%) | 90 (100.0%) |
| Age (years) |  |  |  |
| Mean (SD) | 39.3 (10.1) | 42.0 (12.2) | 40.4 (9.1) |
| 18 to 29 | 33 (18.3%) | 20 (18.5%) | 10 (11.1%) |
| 30 to 39 | 64 (35.6%) | 29 (26.9%) | 36 (40.0%) |
| 40 to 49 | 58 (32.2%) | 33 (30.6%) | 31 (34.4%) |
| 50 to 59 | 17 (9.4%) | 14 (13.0%) | 11 (12.2%) |
| 60 to 69 | 6 (3.3%) | 10 (9.3%) | 2 (2.2%) |
| > 69 | 2 (1.1%) | 2 (1.9%) | 0 (0.0%) |
| Gender |  |  |  |
| Male | 139 (77.2%) | 83 (76.9%) | 82 (91.1%) |
| Female | 41 (22.8%) | 25 (23.2%) | 8 (8.9%) |
| Transmission mode |  |  |  |
| Men who have sex with men | 78 (43.3%) | 50 (46.3%) | 45 (50.0%) |
| Persons with heterosexual contact | 37 (20.6%) | 17 (15.7%) | 14 (15.6%) |
| Persons who inject drugs | 18 (10.0%) | 10 (9.3%) | 12 (13.3%) |
| Persons from high-prevalence countries | 30 (16.7%) | 17 (15.7%) | 11 (12.2%) |
| Other | 0 (0.0%) | 0 (0.0%) | 0 (0.0%) |
| Unknown | 17 (9.4%) | 14 (13.0%) | 8 (8.9%) |
| Country of origin |  |  |  |
| Germany | 117 (65.0%) | 76 (70.4%) | 67 (74.4%) |
| Abroad | 62 (34.4%) | 31 (28.7%) | 22 (24.4%) |
| Unknown | 1 (0.6%) | 1 (0.9%) | 1 (1.1%) |
| CD4 count (cells/µL) |  |  |  |
| Median (IQR) | 153 (40-296) | 181 (85-298) | 214 (119-362) |
| < 50 | 40 (22.2%) | 11 (10.2%) | 8 (8.9%) |
| 50-199 | 47 (26.1%) | 40 (37.0%) | 27 (30.0%) |
| 200-499 | 44 (24.4%) | 29 (26.9%) | 34 (37.8%) |
| > 500 | 16 (8.9%) | 10 (9.3%) | 9 (10.0%) |
| Missing | 33 (18.3%) | 18 (16.7%) | 12 (13.3%) |
| Viral load (copies/mL) |  |  |  |
| Median (IQR) | 45,628 (485-200,000) | 15,000 (350-112,000) | 3,969 (128-139,127) |
| < 50 | 16 (8.9%) | 8 (7.4%) | 11 (12.2%) |
| 50-999 | 26 (14.4%) | 17 (15.7%) | 19 (21.1%) |
| 1,000-9,999 | 16 (8.9%) | 16 (14.8%) | 7 (7.8%) |
| 10,000-99,999 | 29 (16.1%) | 19 (17.6%) | 10 (11.1%) |
| > 100,000 | 57 (31.7%) | 23 (21.3%) | 25 (27.8%) |
| Missing | 36 (20.0%) | 25 (23.2%) | 18 (20.0%) |
| Previous AIDS event^a^ |  |  |  |
| yes | 65 (36.1%) | 32 (29.6%) | 25 (27.8%) |
| no | 115 (63.9%) | 76 (70.4%) | 65 (72.2%) |
| Numbers may not add up to 100% because of rounding  Abbreviations: *PLHIV* People living with HIV *ART* Antiretroviral therapy *SD* Standard deviation  *IQR* Interquartile range  ^a^A previous AIDS event includes AIDS-defining illnesses that were diagnosed after enrolment and before or at the time of ART initiation | | | |

|  | | | |
| --- | --- | --- | --- |
|  | **PLHIV without**  **AIDS event**  ***n* (%)** | **PLHIV who developed an**  **AIDS event within 12 months since ART initiation**  ***n* (%)** | **PLHIV who developed an**  **AIDS event after > 12 months since ART initiation**  ***n* (%)** |
| Total | 15,525 (100.0%) | 668 (100.0%) | 378 (100.0%) |
| Age (years) |  |  |  |
| Mean (SD) | 39.6 (10.9) | 42.6 (11.6) | 40.3 (10.6) |
| 18 to 29 | 2,940 (18.9%) | 76 (11.4%) | 63 (16.7%) |
| 30 to 39 | 5,424 (34.9%) | 220 (32.9%) | 129 (34.1%) |
| 40 to 49 | 4,354 (28.1%) | 196 (29.3%) | 122 (32.3%) |
| 50 to 59 | 2,012 (13.0%) | 112 (16.8%) | 42 (11.1%) |
| 60 to 69 | 651 (4.2%) | 51 (7.6%) | 18 (4.8%) |
| > 69 | 144 (0.9%) | 13 (2.0%) | 4 (1.1%) |
| Sex |  |  |  |
| Male | 12,287 (79.1%) | 557 (83.4%) | 304 (80.4%) |
| Female | 3,238 (20.9%) | 111 (16.6%) | 74 (19.6%) |
| Transmission mode |  |  |  |
| Men who have sex with men | 7,750 (49.9%) | 292 (43.7%) | 173 (45.8%) |
| Persons with heterosexual contact | 2,330 (15.0%) | 118 (17.7%) | 68 (18.0%) |
| Persons who inject drugs | 962 (6.2%) | 36 (5.4%) | 40 (10.6%) |
| Persons from high-prevalence countries | 2,203 (14.2%) | 105 (15.7%) | 58 (15.3%) |
| Other | 98 (0.6%) | 3 (0.5%) | 0 (0.0%) |
| Unknown | 2,182 (14.1%) | 114 (17.1%) | 39 (10.3%) |
| Origin |  |  |  |
| Germany | 10,428 (67.2%) | 450 (67.4%) | 260 (68.8%) |
| Abroad | 4,692 (30.2%) | 201 (30.1%) | 115 (30.4%) |
| Unknown | 405 (2.6%) | 17 (2.5%) | 3 (0.8%) |
| CD4 count (cells/µL) |  |  |  |
| Mean (SD) | 323.7 (239.2) | 155.5 (178.5) | 231.4 (210.7) |
| < 50 | 1,448 (9.3%) | 192 (28.7%) | 60 (15.9%) |
| 50-199 | 3,316 (21.4%) | 185 (27.7%) | 117 (40.0%) |
| 200-499 | 6,463 (41.6%) | 130 (19.5%) | 110 (29.1%) |
| > 500 | 2,774 (17.9%) | 23 (3.4%) | 37 (9.8%) |
| Missing | 1,524 (9.8%) | 138 (20.7%) | 54 (14.3%) |
| Viral load (copies/mL) |  |  |  |
| Mean (SD) | 198,763 (790,174) | 366,662 (1,061,282) | 242,360 (835,526) |
| < 50 | 2,453 (15.8%) | 51 (7.6%) | 40 (10.6%) |
| 50-999 | 2,693 (17.4%) | 71 (10.6%) | 64 (16.9%) |
| 1,000-9,999 | 1,818 (11.7%) | 65 (9.7%) | 39 (10.3%) |
| 10,000-99,999 | 3,129 (20.2%) | 99 (14.8%) | 58 (15.3%) |
| > 100,000 | 3,613 (23.3%) | 233 (34.9%) | 106 (28.0%) |
| Missing | 1,819 (11.7%) | 149 (22.3%) | 71 (18.8%) |
| Previous AIDS event before ART initiation |  |  |  |
| yes | 2,348 (15.1%) | 238 (35.6%) | 122 (32.3%) |
| no | 13,177 (84.9%) | 430 (64.4%) | 256 (67.7%) |
| Numbers may not add up to 100% because of rounding  Abbreviations: *PLHIV* People living with HIV *ART* Antiretroviral therapy *SD* Standard deviation | | | |
