# Supplementary material for: AIDS-defining events among people living with HIV who have been under continuous antiretroviral therapy for more than one year, a German cohort study 1999–2018
Source: Infection. 2024 Feb 21;52(2):637–48. doi: 10.1007/s15010-024-02188-y (PMC10954987; doi:10.1007/s15010-024-02188-y)
Supplement: Supplementary file 2 — Supplementary file2 (DOCX 16 KB) [file 15010_2024_2188_MOESM2_ESM.docx]

**Additional file 2: Tables S2-S3**

| **Table S2 Viral load at the time of the AIDS event among PLHIV who experienced an AIDS-defining event after > 1 year of continuous ART**  Stratified by transmission mode | | | | | |
| --- | --- | --- | --- | --- | --- |
| **Transmission mode** | **MSM**  ***n* (%)** | **HET**  ***n* (%)** | **PWID**  ***n* (%)** | **PHPC**  ***n* (%)** | **Unknown**  ***n* (%)** |
| Total | 173 (100.0%) | 68 (100.0%) | 40 (100.0%) | 58 (100.0%) | 39 (100.0%) |
| Viral load (copies/mL) at the time of the AIDS event^a^ |  |  |  |  |  |
| < 50 | 58 (76.3%) | 21 (70.0%) | 6 (42.9%) | 13 (46.4%) | 14 (66.7%) |
| 50-999 | 6 (7.9%) | 3 (10.0%) | 2 (14.3%) | 5 (17.9%) | 2 (9.5%) |
| 1,000-9,999 | 3 (4.0%) | 1 (3.3%) | 3 (21.4%) | 3 (10.7%) | 1 (4.8%) |
| 10,000-99,999 | 3 (4.0%) | 1 (3.3%) | 2 (14.3%) | 5 (17.9%) | 0 (0.0%) |
| > 100,000 | 6 (7.9%) | 4 (13.3%) | 1 (7.1%) | 2 (7.1%) | 4 (19.1%) |
| Missing^b^ | 97 | 38 | 26 | 30 | 18 |
| Numbers may not add up to 100% because of rounding  Abbreviations: *PLHIV* People living with HIV *ART* Antiretroviral therapy *MSM* Men who have sex with men *HET* Persons with heterosexual contact *PWID* Persons who inject drugs *PHPC* Persons from high-prevalence countries  ^a^As the majority of PLHIV with available viral load values was virally suppressed at the time of the AIDS event, the presentation of a median viral load is not informative  ^b^Due to the high number of missing values, percentages were calculated excluding missing values | | | | | |

| **Table S3 Characteristics at the time of the AIDS event among PLHIV who experienced an AIDS-defining event after > 1 year of continuous ART**  Stratified by years under continuous ART at the time of the AIDS event | | | |
| --- | --- | --- | --- |
| **Years under continuous ART at the time of the AIDS event** | **> 1 to 3 *n* (%)** | **> 3 to 6 *n* (%)** | **> 6 *n* (%)** |
| Total | 180 (100.0%) | 108 (100.0%) | 90 (100.0%) |
| Age (years) at the time of the AIDS event |  |  |  |
| Mean (SD) | 41.1 (10.2) | 46.3 (12.2) | 49.3 (9.3) |
| 18 to 29 | 21 (11.7%) | 4 (3.7%) | 1 (1.1%) |
| 30 to 39 | 65 (36.1%) | 32 (29.6%) | 11 (12.2%) |
| 40 to 49 | 62 (34.4%) | 34 (31.5%) | 36 (40.0%) |
| 50 to 59 | 23 (12.8%) | 20 (18.5%) | 30 (33.3%) |
| 60 to 69 | 7 (3.9%) | 12 (11.1%) | 10 (11.1%) |
| > 69 | 2 (1.1%) | 6 (5.6%) | 2 (2.2%) |
| CD4 count (cells/µL) at the time of the AIDS event |  |  |  |
| Median (IQR) | 302 (127-432) | 262 (82-504) | 397 (139-738) |
| < 50 | 11 (12.2%) | 6 (14.3%) | 4 (9.8%) |
| 50-199 | 23 (25.6%) | 10 (23.8%) | 9 (22.0%) |
| 200-499 | 39 (43.3%) | 15 (35.7%) | 11 (26.8%) |
| > 500 | 17 (18.9%) | 11 (26.2%) | 17 (41.5%) |
| Missing^a^ | 90 | 66 | 49 |
| Viral load (copies/mL) at the time of the AIDS event^b^ |  |  |  |
| < 50 | 59 (66.3%) | 27 (65.9%) | 26 (66.7%) |
| 50-999 | 9 (10.1%) | 5 (12.2%) | 4 (10.3%) |
| 1,000-9,999 | 8 (9.0%) | 0 (0.0%) | 3 (7.7%) |
| 10,000-99,999 | 5 (5.6%) | 3 (7.3%) | 3 (7.7%) |
| > 100,000 | 8 (9.0%) | 6 (14.6%) | 3 (7.7%) |
| Missing^a^ | 91 | 67 | 51 |
| Experienced viral load increase (> 200 copies/mL) before the AIDS event |  |  |  |
| Yes | 30 (16.7%) | 33 (30.6%) | 58 (64.4%) |
| No | 131 (72.8%) | 61 (56.5%) | 29 (32.2%) |
| Never achieved viral  suppression | 19 (10.6%) | 14 (13.0%) | 3 (3.3%) |
| Numbers may not add up to 100% because of rounding  Abbreviations: *PLHIV* People living with HIV *ART* Antiretroviral therapy *SD* Standard deviation  *IQR* Interquartile range  ^a^Due to the high number of missing values in CD4 count and viral load, percentages were calculated excluding missing values  ^b^As the majority of PLHIV with available viral load values was virally suppressed at the time of the AIDS event, the presentation of a median viral load is not informative | | | |
