# Supplementary material for: AIDS-defining events among people living with HIV who have been under continuous antiretroviral therapy for more than one year, a German cohort study 1999–2018
Source: Infection. 2024 Feb 21;52(2):637–48. doi: 10.1007/s15010-024-02188-y (PMC10954987; doi:10.1007/s15010-024-02188-y)
Supplement: Supplementary file 3 — Supplementary file3 (DOCX 16 KB) [file 15010_2024_2188_MOESM3_ESM.docx]

| **Additional file 3: Table S4**  **Table S4 Five most frequently observed AIDS-defining illnesses among PLHIV who experienced an AIDS-defining event after > 1 year of continuous ART**  Stratified by years under continuous ART at the time of the AIDS event, transmission mode, CD4 count at the time of the AIDS event, and viral load at the time of the AIDS event | |  |
| --- | --- | --- |
|  | **AIDS-defining illness, *n* (%)** | |
| **Years under continuous ART at the time of the AIDS event** |  | |
| > 1 to 3 | Total^a^ 195 (100.0%) | |
|  | Oesophageal candidiasis 27 (13.8%) | |
|  | Wasting syndrome 21 (10.8%) | |
|  | Pneumocystis-jirovecii-pneumonia 19 (9.7%) | |
|  | Extrapulmonary tuberculosis 18 (9.2%) | |
|  | Pulmonary tuberculosis 16 (8.2%) | |
|  |  | |
| > 3 to 6 | Total^a^ 121 (100.0%) | |
|  | Pneumocystis-jirovecii-pneumonia 16 (13.2%) | |
|  | Oesophageal candidiasis 15 (12.4%) | |
|  | Wasting syndrome 15 (12.4%) | |
|  | HIV encephalitis 14 (11.6%) | |
|  | HSV ulcers 12 (9.9%) | |
|  |  | |
| > 6 | Total^a^ 100 (100.0%) | |
|  | Oesophageal candidiasis 19 (19.0%) | |
|  | HIV encephalitis 13 (13.0%) | |
|  | Wasting syndrome 11 (11.0%) | |
|  | Pneumocystis-jirovecii-pneumonia 8 (8.0%) | |
|  | Atypical mycobacteria 8 (8.0%) | |
| **Transmission mode^b^** |  | |
| Men who have sex with  men | Total^a^ 191 (100.0%) | |
|  | HSV ulcers 21 (11.0%) | |
|  | Wasting syndrome 21 (11.0%) | |
|  | Oesophageal candidiasis 20 (10.5%) | |
|  | Non-Hodgkin lymphoma 19 (9.9%) | |
|  | HIV encephalitis 19 (9.9%) | |
|  |  | |
| Persons with heterosexual  contact | Total^a^ 78 (100.0%) | |
|  | Pneumocystis-jirovecii-pneumonia 13 (16.7%) | |
|  | Oesophageal candidiasis 11 (14.1%) | |
|  | Wasting syndrome 9 (11.5%) | |
|  | Cerebral toxoplasmosis 6 (7.7%) | |
|  | Atypical mycobacteria 4 (5.6%) | |
|  |  | |
| Persons who inject drugs | Total^a^ 46 (100.0%) | |
|  | Oesophageal candidiasis 13 (28.3%) | |
|  | Wasting syndrome 12 (26.1%) | |
|  | Pneumocystis-jirovecii-pneumonia 5 (10.9%) | |
|  | HIV encephalitis 3 (6.5%) | |
|  | Recurrent pneumonia 3 (6.5%) | |
|  |  | |
| Persons from high-  prevalence countries | Total^a^ 60 (100.0%) | |
|  | Extrapulmonary tuberculosis 15 (25.0%) | |
|  | HIV encephalitis 11 (18.3%) | |
|  | Oesophageal candidiasis 8 (13.3%) | |
|  | Pulmonary tuberculosis 7 (11.7%) | |
|  | HSV ulcers 7 (11.7%) | |
| **CD4 count (cells/µL) at the time of the AIDS event** |  | |
| < 50 | Total^a^ 32 (100.0%) | |
|  | Oesophageal candidiasis 10 (31.3%) | |
|  | Wasting syndrome 5 (15.6%) | |
|  | Pneumocystis-jirovecii-pneumonia 4 (12.5%) | |
|  | Cerebral toxoplasmosis 3 (9.4%) | |
|  | Cytomegalovirus 3 (9.4%) | |
|  |  | |
| 50-199 | Total^a^ 49 (100.0%) | |
|  | Oesophageal candidiasis 9 (18.4%) | |
|  | Pneumocystis-jirovecii-pneumonia 8 (16.3%) | |
|  | Wasting syndrome 5 (10.2%) | |
|  | Cerebral toxoplasmosis 4 (8.2%) | |
|  | Cytomegalovirus 4 (8.2%) | |
|  |  | |
| 200-499 | Total^a^ 67 (100.0%) | |
|  | HIV encephalitis 12 (17.9%) | |
|  | Wasting syndrome 8 (11.9%) | |
|  | Oesophageal candidiasis 7 (10.4%) | |
|  | Pulmonary tuberculosis 7 (10.4%) | |
|  | Pneumocystis-jirovecii-pneumonia 6 (9.0%) | |
|  |  | |
| > 500 | Total^a^ 47 (100.0%) | |
|  | HSV ulcers 8 (17.0%) | |
|  | Wasting syndrome 5 (10.6%) | |
|  | Kaposi’s sarcoma 5 (10.6%) | |
|  | Extrapulmonary tuberculosis 4 (8.5%) | |
|  | HIV encephalitis 4 (8.5%) | |
|  |  | |
| Missing | Total^a^ 221 (100.0%) | |
|  | Oesophageal candidiasis 32 (14.5%) | |
|  | Wasting syndrome 24 (10.9%) | |
|  | Pneumocystis-jirovecii-pneumonia 23 (10.4%) | |
|  | HIV encephalitis 21 (9.5%) | |
|  | Non-Hodgkin lymphoma 20 (9.0%) | |
| **Viral load (copies/mL) at the time of the AIDS event** |  | |
| < 50 | Total^a^ 119 (100.0%) | |
|  | Pneumocystis-jirovecii-pneumonia 14 (11.8%) | |
|  | Wasting syndrome 13 (10.9%) | |
|  | HSV ulcers 13 (10.9%) | |
|  | Oesophageal candidiasis 12 (10.1%) | |
|  | HIV encephalitis 12 (10.1%) | |
|  |  | |
| 50-999 | Total^a^ 21 (100.0%) | |
|  | Oesophageal candidiasis 5 (23.8%) | |
|  | HIV encephalitis 5 (23.8%) | |
|  | Pneumocystis-jirovecii-pneumonia 2 (9.5%) | |
|  | Wasting syndrome 2 (9.5%) | |
|  | Cytomegalovirus 2 (9.5%) | |
|  |  | |
| 1,000-9,999 | Total^a^ 12 (100.0%) | |
|  | HIV encephalitis 2 (16.7%) | |
|  | Wasting syndrome 2 (16.7%) | |
|  | Progressive multifocal leukoencephalopathy 2 (16.7%) | |
|  | Extrapulmonary tuberculosis 1 (8.3%) | |
|  | Pneumocystis-jirovecii-pneumonia 1 (8.3%) | |
|  |  | |
| 10,000-99,999 | Total^a^ 11 (100.0%) | |
|  | HIV encephalitis 2 (18.2%) | |
|  | Oesophageal candidiasis 2 (18.2%) | |
|  | Cerebral toxoplasmosis 1 (9.1%) | |
|  | Wasting syndrome 1 (9.1%) | |
|  | Extrapulmonary tuberculosis 1 (9.1%) | |
|  |  | |
| > 100,000 | Total^a^ 22 (100.0%) | |
|  | Oesophageal candidiasis 6 (27.3%) | |
|  | Wasting syndrome 5 (18.5%) | |
|  | Cerebral toxoplasmosis 3 (13.6%) | |
|  | HIV encephalitis 2 (9.1%) | |
|  | Pneumocystis-jirovecii-pneumonia 2 (9.1%) | |
|  |  | |
| Missing | Total^a^ 231 (100.0%) | |
|  | Oesophageal candidiasis 36 (15.6%) | |
|  | Wasting syndrome 24 (10.4%) | |
|  | Pneumocystis-jirovecii-pneumonia 23 (10.0%) | |
|  | Non-Hodgkin lymphoma 20 (8.7%) | |
|  | HIV encephalitis 19 (8.2%) | |
|  |  | |

Abbreviations: *PLHIV* People living with HIV *ART* Antiretroviral therapy

^a^The number of recorded AIDS-defining illnesses exceeds the number of first AIDS-defining events since

an AIDS event can comprise more than one AIDS-defining illness if these were recorded in the same month

^b^Transmission groups “other” and “unknown” are not presented due to sparse data and limited interpretability
