# Supplementary material for: AIDS-defining events among people living with HIV who have been under continuous antiretroviral therapy for more than one year, a German cohort study 1999–2018
Source: Infection. 2024 Feb 21;52(2):637–48. doi: 10.1007/s15010-024-02188-y (PMC10954987; doi:10.1007/s15010-024-02188-y)
Supplement: Supplementary file 4 — Supplementary file4 (DOCX 15 KB) [file 15010_2024_2188_MOESM4_ESM.docx]

**Additional file 4: Tables S5-S9**

| **Table S5 Bootstrap replication inclusion frequencies of potential predictors for an AIDS-defining event among PLHIV** **after > 1 year of continuous ART**  Only including person-time of PLHIV between 1999 and 2009 | | | |  |
| --- | --- | --- | --- | --- |
| **Covariate** | **Absolute inclusion frequency^a^** | **Relative inclusion frequency** | |  |
|  |  | |  | |
| Previous AIDS event before ART initiation | 495 | | 99.0% | |
| CD4 count (cells/µL) at ART initiation | 478 | | 95.6% | |
| Age at ART initiation | 190 | | 38.0% | |
| Viral load (copies/mL) at ART initiation | 172 | | 34.4% | |
| Transmission mode^b^ | 79 | | 15.8% | |
| Gender | 73 | | 14.6% | |
| Country of origin | 71 | | 14.2% | |
|  |  | |  | |
| Abbreviations: *PLHIV* People living with HIV *ART* Antiretroviral therapy  ^a^Absolute inclusion frequencies after 500 bootstrap replications of the original data set  ^b^Transmission groups “other” and “unknown” were excluded from the analyses due to sparse data and limited interpretability | | | | |

| **Table S6 Bootstrap replication inclusion frequencies of potential predictors for an AIDS-defining event among PLHIV after > 1 year of continuous ART**  Only including person-time of PLHIV between 2010 and 2018 | | | |  |
| --- | --- | --- | --- | --- |
| **Covariate** | **Absolute inclusion frequency^a^** | **Relative inclusion frequency** | |  |
|  |  | |  | |
| Transmission mode^b^ | 275 | | 55.0% | |
| CD4 count (cells/µL) at ART initiation | 240 | | 48.0% | |
| Viral load (copies/mL) at ART initiation | 229 | | 45.8% | |
| Previous AIDS event before ART initiation | 181 | | 36.2% | |
| Gender | 172 | | 34.4% | |
| Country of origin | 69 | | 13.8% | |
| Age at ART initiation | 66 | | 13.2% | |
|  |  | |  | |
| Abbreviations: *PLHIV* People living with HIV *ART* Antiretroviral therapy  ^a^Absolute inclusion frequencies after 500 bootstrap replications of the original data set  ^b^Transmission groups “other” and “unknown” were excluded from the analyses due to sparse data and limited interpretability | | | | |

| **Table S7 Bootstrap replication inclusion frequencies of potential predictors for an AIDS-defining event among PLHIV after > 1 year of continuous ART (transmission groups included as separate binary variables)^a^** | | | |  |
| --- | --- | --- | --- | --- |
| **Covariate** | **Absolute inclusion frequency^b^** | **Relative inclusion frequency** | |  |
|  |  | |  | |
| CD4 count (cells/µL) at ART initiation | 500 | | 100.0% | |
| Previous AIDS event before ART initiation | 498 | | 99.6% | |
| People who inject drugs | 407 | | 81.4% | |
| People with heterosexual contact | 156 | | 31.2% | |
| Country of origin | 112 | | 22.4% | |
| People from high-prevalence countries | 103 | | 20.6% | |
| Viral load (copies/mL) at ART initiation | 64 | | 12.8% | |
| Gender | 58 | | 11.6% | |
| Age at ART initiation | 54 | | 10.8% | |
|  |  | |  | |
| Abbreviations: *PLHIV* People living with HIV *ART* Antiretroviral therapy  ^a^Men who have sex with men served as the reference category and were not included as a variable for collinearity reasons. Transmission groups “other” and “unknown” were excluded from the analyses due to sparse data and limited interpretability.  ^b^Absolute inclusion frequencies after 500 bootstrap replications of the original data set | | | | |

| **Table S8 Bootstrap replication inclusion frequencies of potential predictors for an AIDS-defining event among PLHIV after > 1 year of continuous ART (transmission groups included as separate binary variables)^a^**  Only including person-time of PLHIV between 1999 and 2009 | | | |  |
| --- | --- | --- | --- | --- |
| **Covariate** | **Absolute inclusion frequency^b^** | **Relative inclusion frequency** | |  |
|  |  | |  | |
| Previous AIDS event before ART initiation | 496 | | 99.2% | |
| CD4 count (cells/µL) at ART initiation | 483 | | 96.6% | |
| People from high-prevalence countries | 214 | | 42.8% | |
| Viral load (copies/mL) at ART initiation | 194 | | 38.8% | |
| Age at ART initiation | 156 | | 31.2% | |
| People with heterosexual contact | 141 | | 28.2% | |
| Country of origin | 134 | | 26.8% | |
| Gender | 77 | | 15.4% | |
| People who inject drugs | 53 | | 10.6% | |
|  |  | |  | |
| Abbreviations: *PLHIV* People living with HIV *ART* Antiretroviral therapy  ^a^Men who have sex with men served as the reference category and were not included as a variable for collinearity reasons. Transmission groups “other” and “unknown” were excluded from the analyses due to sparse data and limited interpretability.  ^b^Absolute inclusion frequencies after 500 bootstrap replications of the original data set | | | | |

| **Table S9 Bootstrap replication inclusion frequencies of potential predictors for an AIDS-defining event among PLHIV after > 1 year of continuous ART (transmission groups included as separate binary variables)^a^**  Only including person-time of PLHIV between 2010 and 2018 | | | |  |
| --- | --- | --- | --- | --- |
| **Covariate** | **Absolute inclusion frequency^b^** | **Relative inclusion frequency** | |  |
|  |  | |  | |
| People from high-prevalence countries | 299 | | 59.8% | |
| CD4 count (cells/µL) at ART initiation | 243 | | 48.6% | |
| Viral load (copies/mL) at ART initiation | 232 | | 46.4% | |
| Previous AIDS event before ART initiation | 189 | | 37.8% | |
| Gender | 182 | | 36.4% | |
| People who inject drugs | 164 | | 32.8% | |
| People with heterosexual contact | 136 | | 27.2% | |
| Country of origin | 53 | | 10.6% | |
| Age at ART initiation | 50 | | 10.0% | |
|  |  | |  | |
| Abbreviations: *PLHIV* People living with HIV *ART* Antiretroviral therapy  ^a^Men who have sex with men served as the reference category and were not included as a variable for collinearity reasons. Transmission groups “other” and “unknown” were excluded from the analyses due to sparse data and limited interpretability.  ^b^Absolute inclusion frequencies after 500 bootstrap replications of the original data set | | | | |
